# Diurnal Variation in Physiological and Immune Responses to Endurance Sport in Highly Trained Runners in a Hot and Humid Environment

**DOI:** 10.1155/2018/3402143

**Published:** 2018-05-09

**Authors:** B. Boukelia, E. C. Gomes, G. D. Florida-James

**Affiliations:** School of Applied Sciences, Edinburgh Napier University, Edinburgh, UK

## Abstract

**Purpose:**

The purpose of this study was to investigate the physiological and immunological response of highly trained runners to an intense bout of exercise performed at two different times of day, in a hot, humid environment.

**Methods:**

Using a crossover randomized design, 13 highly trained runners (range $\dot{V}O_{2}\max$ 64–79 ml·kg^−1^ min^−1^) performed a 10 km time trial run in hot (28°C) and humid conditions (70%), at 2 different times of day (09:00 hs and 18:00 hs). Venous blood samples were taken to determine WBCs (white blood cells), IL-6 (interleukin-6), CC16 (club cell protein 16), and HSP70 (heat shock protein-70) concentrations. Upper respiratory tract inflammation was additionally assessed using a nasal lavage procedure.

**Results:**

A significant diurnal difference (*p* < 0.05) was found for core body temperature, total WBC, and neutrophil and lymphocyte concentrations with higher values at 18:00 hs. A phase response in IL-6, HSP70, WBC, neutrophil, lymphocyte, and CC16 was noted, being more pronounced at 18:00 hs, whilst core body temperature and HR phase responses were more pronounced at 09:00 hs.

**Conclusion:**

In hot and humid conditions, athletes may wish to consider, when possible, racing and particularly training in the morning where the least homeostatic perturbation occurs.

## 1. Introduction

Bright light is the principal zeitgeber (time giver) for the biological clock in mammals, including humans. Maintenance of regular circadian timing is important to human health and well-being, and physical exercise has been shown to entrain circadian rhythms and can cause phase shifting [1]. The effect of time of the day on performance has been well established with early morning nadirs and late afternoon peaks [2]. This diurnal rhythm can be influenced by several factors, including exposure to hot and humid environmental conditions, which in turn are factors that can affect sports performance [3, 4].

There are substantial physiological, immunological, and psychological changes that occur in an athlete during exercise in the heat compared with the habitual environment [5]. These changes include thermoregulatory alterations in the circulatory and endocrine systems, cardiovascular strain, glycogen depletion, and increased metabolite accumulation [6, 7]. Many interrelated physiological processes work together to maintain muscular function, sustain central blood pressure, regulate fluid volume, and maintain the body temperature within normal limits [8].

Several of these exacerbated physiological demands may be responsible for mobilising a larger number of white blood cells (WBCs) into the circulation when exposed to the heat [9]. It is already known, for example, that the amount that interleukin-6 (IL-6) increases is correlated to exercise intensity, duration, muscle mass, and the power involved in the mechanical work [10]. Moreover, it has been suggested that the release of IL-6 in exercise is related to the occurrence of muscle damage [11, 12]. Heat shock protein (HSP70) can be treated as a part of the stress response as well [13], which increases when human cells are exposed to elevated temperatures or to other stress types such as exercise. A perception exists that some athletes, especially those engaging in prolonged intense exercise and competition, such as running, cycling, and swimming, may show an increased rate of upper respiratory tract infection (URTI) [14, 15], owing to a compromised immune system. Moderate exercise enhances the athlete's immunity, but high-intensity prolonged exercise impairs, temporarily, immune competence [16, 17].

The existence of circadian rhythmicity in the human immune system has been recorded for some time [18–21]. It has been reported in Haus and Smolensky [19] that the circulation of the WBCs involved in the defence of the human body shows high-amplitude circadian rhythmicity, being lower in the morning and higher in the afternoon. Additionally, a diurnal variation has been observed in IL-6, with a decrease at 12:00 hs, 16:00 hs, and 20:00 hs compared to 07:00 hs during high-force eccentric resistance exercise using the elbow flexor muscles [20]. HSP70 also shows a diurnal variation which strongly correlates with core body temperature, with a maximum reported in the evening and a nadir in the early morning [13]. Moreover, club cell protein 16 (CC16) concentration, which plays an important role in protecting the respiratory tract against inflammation and oxidative stress and therefore is a suitable biomarker of lung inflammation and injury, shows a diurnal variation, with a decrease during daytime hours [22–24].

Elite athletes not only compete against one another but also compete against tough environmental conditions. Challenging environmental conditions such as heat or cold during racing or training phases pose particular risks to an athlete's health. In modern sports history, the Olympic Games, World Championships, and major races have drawn attention to a number of climatic effects on running performance [25]. The summer competition calendar events such as the Olympics, Outdoor World Championships, and some major road races have all been held in hot and humid conditions.

Thus, intense exercise in the heat can overload the body's ability to react appropriately to the stress imposed on athletes, and the result of this can be hyperthermia, dehydration, a decrease in physical and mental performance, and even heat stroke and immune depletion [8]. Identifying circadian factors influencing athletic performance is often difficult because of the interaction between endogenous and exogenous components which contribute to overall performance, and in addition, any time-of-day effect of exercise on inflammatory responses remains vague.

The aim of this study was to evaluate the impact on physiological and immunological variables of highly trained runners, completing a 10 km time trial run in a hot and humid environment at two time points within the diurnal day. We, therefore, hypothesised that (1) running performance will reflect a diurnal variation, with peak performance occurring in the evening; (2) there will be a circadian influence on immunological and physiological response to the exercise stress, in addition to the response to the hot and humid condition (28°C and relative humidity of 70%).

## 2. Materials and Method

### 2.1. Study Population

British or European runners are exposed to a range of environmental conditions they are unaccustomed to as they compete around the world (such as IAAF Outdoor World Championships in Doha, 2019). Therefore, investigations into the impact of different environmental conditions are required to understand their impact on athletes. A running trial in hot and humid conditions was selected for investigation. This study was approved by the University Ethics Committee and was conducted in accordance with the guidelines of the Declaration of Helsinki. Anthropometric measurements were taken prior to the trial including height and mass. Thirteen highly trained runners (range $\dot{V}O_{2}\max$ 64–79 ml·kg^−1^ min^−1^; age 33 ± 5 yr) were enrolled in this study. All participants trained daily (covering 80–120 km a week), with some participants training twice daily, with the main training session for athletes is in the evening. All participants were healthy, injury free, not on medication for inflammatory conditions, and without infection within the three weeks prior to the study. The participants in the study all lived in Scotland, UK, and were not accustomed to the hot and humid conditions employed in this study.

### 2.2. Exercise Protocol

A 10 km treadmill time trial was performed under controlled environmental conditions: 28°C, 70% relative humidity in an environmental chamber. The chamber had a complete volume change (76 m^3^) every 15 min during the trials and had been recently serviced and calibrated by the manufacturers (Weiss Technik, UK). Trials were conducted on two separate occasions, separated by at least a week, at two different times of the day (09:00 hs and 18:00 hs). Heart rate (Polar Electro, Finland) was recorded pretrial, each 1 km during the trial, posttrial, and 1 h posttrial, whilst running speed was recorded at the end of each km. The athletes had free control of their running speed, without having access to the value of the actual treadmill speed.

### 2.3. Body Temperature (Gastrointestinal Temperature)

Core body temperature was measured by ingesting a telemetric temperature sensor (ThermoDot, USA). Participants were required to swallow the temperature pills two hours prior to the trial. Measurements were taken at pretrial, each 1 km during the trial, posttrial, and 1 h posttrial.

### 2.4. Blood Sample, BP, and Lung Function

Lung function tests, blood sampling, blood pressure (Wrist, Omron, 5-1, Japan), and anthropometric measurements were conducted pre, post, and 1 h posttrial. Lung function tests including forced vital capacity (FVC and used to help determine both the presence and severity of lung diseases), forced expiratory volume in 1 second (FEV1 and used to calculate Tiffeneau-Pinelli index), peak expiratory flow (PEF), and forced expiratory flow_25–75%_ (FEF25–75) were measured using a spirometer (Compact II: Type C, Vitalograph Ltd., UK).

Blood samples were collected in a 2 ml tube (BD Vacutainer SST™) and differential counts carried out using an automated cell counter (Sysmex, XS-1000i, USA). The numbers and types of different cells within the blood were determined by the cell counting component and automatically saved ready for review (RBC, WBC, and platelet); the samples were run in triplicate and the mean values were obtained.

### 2.5. Biochemical Analyses

For the determination of CC16, HSP70, and IL-6, blood samples were collected in a 6 ml tube (BD Vacutainer SST) and immediately centrifuged for 15 min at 1000*g* at room temperature (Mistral 2000R, Sanyo, Leicester, UK), and the serum was separated, aliquoted, and frozen at −80°C until analysis.

All the measurements were performed using commercially available enzyme-linked immunosorbent assay (ELISA) kits (R&D Systems Europe Ltd.) in accordance with the manufacturer's instructions.

### 2.6. Nasal Lavage Method

The method of Naclerio et al. [26] was modified to increased cell concentrations in the sample based on previous pilot work in our lab. The temperature of the saline solution was increased from 37°C to 40°C, and a gentle massage of the nose was performed prior to conducting the test. These changes increased the amount of cell release from the nostril during sampling.

At rest, prior to the testing, and directly after finishing the 10 km trial, nasal lavage was performed. Participants performed a gentle massage to the nose immediately before the procedure. Saline water was prewarmed to 40°C. Participants tilt their head backwards to a 45° angle, elevating their palate and closing the nasopharynx. Then 5 ml of pre-warmed (40°C) the sterile saline solution was introduced into the nostril and held for a duration of 10 seconds. The participant then brought their head forward and expired the lavage into a polyamide gauze-filtered funnel (size 100 mm) that separated the mucus from the solution, and the samples were collected at the base in a 15 ml centrifuge tube. The nasal lavage procedure was performed again with the other nostril. The volume collected from both nostrils was stored in the same tube; the total volume collected was recorded and immediately placed on ice until analysis.

### 2.7. Statistical Analysis

Prior to statistical analysis, all data were checked for normality. Data were analysed using a two-way repeated measures ANOVA with Bonferroni-adjusted post hoc tests to determine the difference at which time point the diurnal variation occurred and the difference between time points of the trial (SPSS20 Statistical Software, IBM, UK). This analysis revealed the global effect of time of day which includes resting state, the global effect of the environmental conditions, and the effect of the interaction between time of day and the environmental conditions which include posttrial and 1 h posttrial state. A paired sample *t*-test was used to determine any diurnal variation in stature. Statistical significance was accepted at *p* < 0.05. Results are represented as mean values ± standard deviation (SD).

## 3. Results

### 3.1. Diurnal Variation in Anthropometric Measurement and Physiological Performance Variables

There was a significant diurnal variation in stature (*p* = 0.01, *t*[12] = 3.87). Cohen's *d* for this test was 0.281 (which can be described as small; Table 1) where the participants were taller by a mean of 1.46 cm in the morning trial compared to the evening trial. Participant's fluid losses show neither diurnal variation nor trial time point differences (1.02 kg in the morning trial and 0.79 kg in the evening trial).

### 3.2. Performance Variables

**Figure 1:**
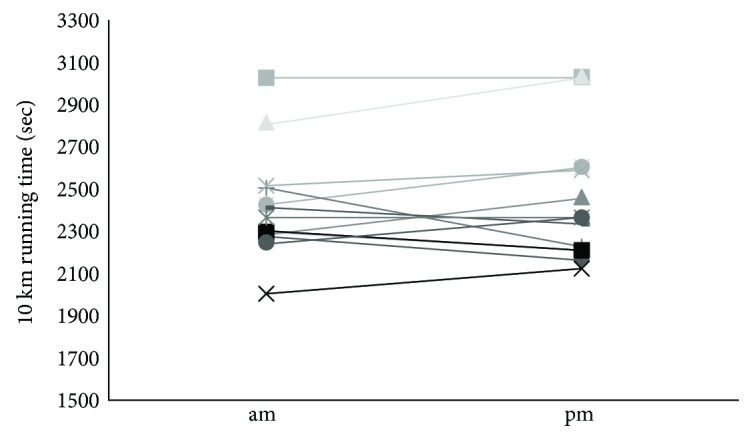
The 13-individual time to complete the running trial at 09:00 hs and 18:00 hs. No significant diurnal variation was observed.

There was no significant difference between 09:00 hs and 18:00 hs trial running performance. However, the mean running time to complete the 10 km trial at 09:00 hs was 19 seconds faster than at 18:00 hs (Figure 1).

### 3.3. Diurnal Variation in Core Body Temperature

**Figure 2:**
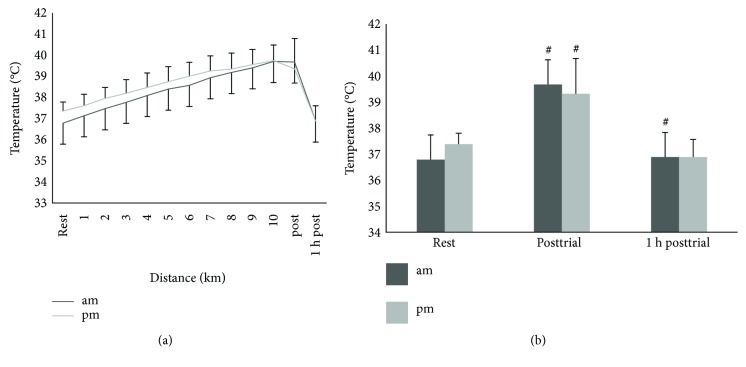
(a) Core body temperature at rest, posttrial, and 1 h posttrial at both times of the day. (b) Core body temperature measured throughout the trials. # denotes a significant difference between trial time points (*p* < 0.05); values are mean ± SD.

There was a significant diurnal variation found between trials for core body temperature (*F*_2,12_ = 2.02, *p* = 0.02, and partial *η*^2^ = 0.16), with a Bonferroni-adjusted post hoc test revealing that core body temperature was higher in the evening (Figures 2(a) and 2(b)). Core body temperature showed a significant phase response (response to a stimulus at a certain time point within a circadian rhythm) as a result of the environmental condition, (*F*_1,10_ = 55.1, *p* < 0.01, and partial *η*^2^ = 0.8). The athlete core body temperature response to the heat and humidity stimulus was higher in the morning by 0.52°C (*p* = 0.01). Additionally, the rate of heat loss was more pronounced in the morning trial compared to the evening trial (*F*_1,10_ = 76.1, *p* < 0.01, and partial *η*^2^ = 0.8), (+0.09°C at 09:00 hs and −1.06°C at 18:00 hs) (Figures 2(a) and 2(b)). There was a significant difference between time trial points (*F*_2,20_ = 62.9, *p* < 0.01, and partial *η*^2^ = 0.9), with Bonferroni-adjusted post hoc tests revealing that the core body temperature had significantly changed at both times of the day (*p* = 0.01).

### 3.4. Diurnal Variation in Peak and Resting HR

There was no diurnal difference between trials in HR. However, a significant difference between trial time points was observed at both times of the day (*F*_2,24_ = 875, *p* < 0.01, and partial *η*^2^ = 0.9). HR displayed a significant phase response to the environmental condition (*F*_1,12_ = 1356, *p* < 0.01, and partial *η*^2^ = 0.9), with HR response more pronounced in the morning trial compared to the evening trial. Mean speed, resting HR, and peak HR (Table 2) did not show a significant difference between the two trials.

Physiological variables including HR and core body temperature appeared to show that the morning trial had a larger effect on these variables compared to the evening trial.

### 3.5. Diurnal Variation in MAP, RBC, HCT, HGB, and Lung Function Measures

No diurnal variation was found in either MAP, RBC, HCT, HGB, or PLT (*p* > 0.05). However, PLT concentration showed a significant difference between measured time points (*F*_2,22_ = 18.9, *p* < 0.01, and partial *η*^2^ = 0.6). Bonferroni-adjusted post hoc tests revealed that PLT was significantly affected by exercise and environmental condition at both times (*p* = 0.01). In addition, lung function variables (FVC, PEF, FEV1, FEF_25–75%_, and FEV1/FVC ratio) did not show diurnal variation.

### 3.6. Diurnal Variation in White Blood Cells (WBCs)

**Figure 3:**
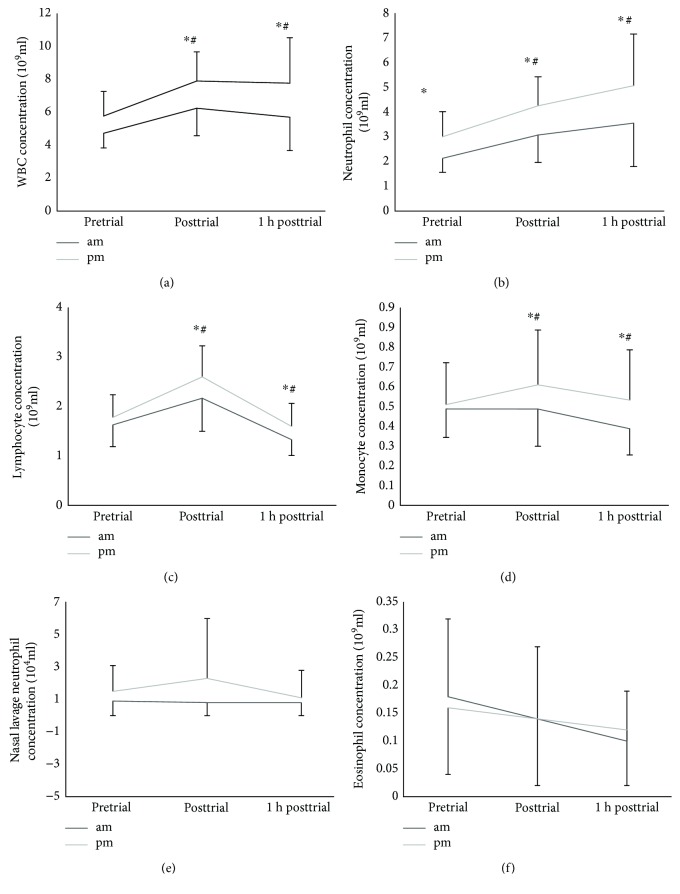
(a) Total white blood cells, (b) neutrophil (c) lymphocyte, (d) monocytes, (e) nasal lavage neutrophil, and (f) eosinophil. ∗ indicates significantly different (*p* < 0.05) and diurnal variation (*p* < 0.05), and # indicates a time point significant different (*p* < 0.05). Values are mean ± SD.

A significant diurnal difference was found in total WBC, neutrophil, lymphocyte, and monocytes concentrations: the effect sizes (ES) for WBC were 0.95 (95% CI 5.02–6.62 and 5.28–7.22; *p* < 0.01). In both trials, WBC showed a significant difference between time points (*p* < 0.01). In addition, WBC appeared to be more affected by the hot and humid conditions in the evening trial (*p* = 0.01) (Figure 3(a)). Neutrophil concentration showed a significant difference (*p* < 0.02) between time points in both trials. Neutrophil effect sizes (ES) were 0.65 (95% CI 2.73–3.95 and 2.99–4.41, *p* < 0.002) (Figure 3(b)). Lymphocytes displayed a significant difference between measured time points (*p* < 0.01: *p* = 0.01), in both trials. Lymphocyte ES were 0.51 (95% CI 1.49–1.96 and 1.72–2.31, *p* < 0.002), (Figure 3(c)). In addition, monocyte concentration in both trials showed a significant difference between measured time points (*p* < 0.01). Monocyte ES were 0.34 (95% CI 0.33–0.49 and 0.36–0.64, *p* < 0.03) (Figure 3(d)). Neither nasal lavage neutrophil (e) nor eosinophil (f) concentrations were affected by time of the day. The phase response of WBC, neutrophil, and monocyte concentrations to the environmental condition and exercise was more pronounced in the evening trial (Figures 3(a), 3(b), and 3(d)) (*p* = 0.01).

Immunological parameters including WBC, neutrophil, and lymphocyte demonstrated a higher evening phase response to exercise in the hot and humid condition.

### 3.7. Diurnal Variation in Plasma CC16, HSP70, and IL-6

**Figure 4:**
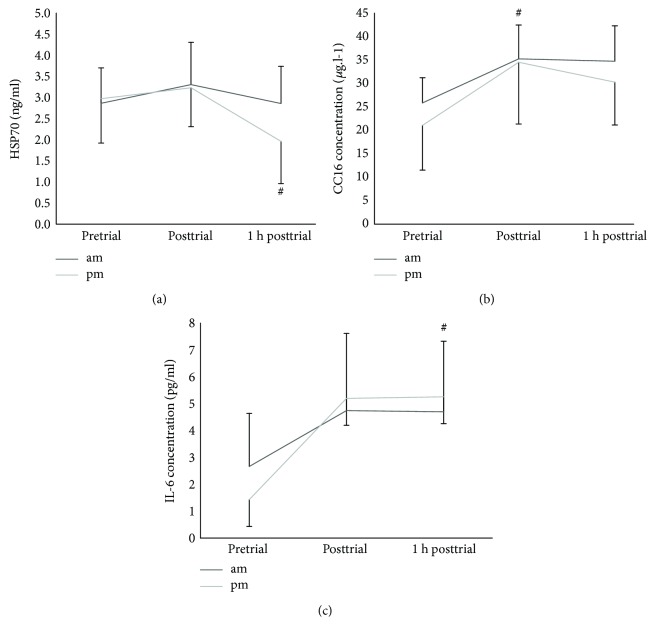
(a) HSP70, (b) CC16, and (c) IL-6. # indicates a time point significant difference (*p* < 0.05). Values are mean ± SD.

Neither plasma CC16, HSP70, nor IL-6 showed a significant diurnal variation between trials (Figure 4). However, there was a time point interval difference in HSP70 (*F*_2,20_ = 11.2, *p* < 0.001, and partial *η*^2^ = 0.5), with Bonferroni-adjusted post hoc tests revealing that plasma HSP70 was more affected during the evening trial (*p* = 0.004). There was a significant increase in plasma CC16 immediately posttrial for both times of the day (*p* < 0.01); Bonferroni-adjusted post hoc tests revealed that plasma CC16 was more affected at the 3 time point intervals at 09:00 hs than at the 18:00 hs trial. Analysis additionally showed that there was a significant difference in measured plasma IL-6 values between time points (*F*_2,18_ = 24.4, *p* < 0.01, and partial *η*^2^ = 0.7) with Bonferroni-adjusted post hoc tests further revealing that IL-6 levels were more affected at 09:00 hs from pretrial to posttrial than at 18:00 hs trial (*p* = 0.01) (Figure 4(c)). In addition, an evening phase response in IL-6, HSP70, and CC16 was observed.

## 4. Discussion

Our findings add to the existing literature by defining a major impact of circadian rhythm physiology and immunology on exercise performance [3, 10, 27]. A greater phase response in both core body temperature and HR causing a higher physiological strain in the morning trial was evident; whereas, immunological markers including IL-6, HSP70, WBC, neutrophil, lymphocyte, and CC16 were more affected in the evening trial.

Mean running times were not statistically different, between the two time points despite the mean morning trial time being faster by 19 seconds compared to the evening trial. This result contradicts previous research where a significant difference in time-of-day variation in running performance has been reported [27, 28]. All participants (100%) in this study ran slower than they were accustomed to, suggesting that environmental temperature and humidity changes were the main factors influencing their performance and importantly masking any effect of the time of day. In addition, unaccustomed environmental conditions have had a direct effect on core body temperature and HR, with a significant phase response evident in the morning compared to the evening. This may be linked directly to the athlete's greater ability to remove heat load in the morning compared to the evening, a phenomenon explained by the body's heat gain mode during the morning compared to the evening when the body exhibits slow heat gain so that heat loss is more pronounced [29]. The current data suggest that morning exercise exerts higher physical demands on the individual.

Contradicting previous findings, plasma CC16, lung function measures, and nasal lavage neutrophil concentration did not display circadian variation [30, 31]. Again, the overriding effect of the heat and humidity compared to alterations caused by diurnal variation could be masking results. However, a significant difference between trial time points occurred, with an increase in CC16 levels posttrials, irrespective of time of day; this is in agreement with previous findings [22]. In addition, the effect of warm and humid environmental condition and exercise on the increase on CC16 was limited (lower) compared to another similar study conducted by the same authors in a cold environmental condition [32]. Environmental conditions are known to impact the degree of airway epithelial disruption during high levels of exercise, in particular, warm humid air inhalation appears to limit the airway injury and epithelial cell perturbation compared to cold dry air [32, 33]. This finding highlights the potential for exercise, when performed at high intensity in hot and humid conditions, to limit the airway epithelial cell disruption and hence could be employed as a strategy to minimise dehydration stress to the airways and therefore protect the long-term respiratory health of elite winter athletes [33].

Unlike RBC total concentration, HGB, HCT, and platelet numbers, there was an evident diurnal rhythmicity in total WBC, neutrophil, lymphocyte, and monocyte concentrations that was higher in the evening compared to the morning at all time points in the trials. These variables also displayed a response to the exercise/environment stress, including the classic lymphocyte biphasic response to an intense bout of exercise [16]. There did appear to be a phase response in the IL-6 measures, however, with a greater increase in the evening trial compared to morning. The altered pattern of IL-6 suggests that the combination of exercise in the evening time, as well as the hot and humid environment, elicited greater circulating numbers of IL-6 compared to the morning. This elevated evening IL-6 concentration could be due to an increase in total white blood cell concentration (including neutrophil and lymphocyte) in the evening as these cells are known to express IL-6 [34].

We know that in postintense exercise, there is an associated open window to immune perturbation with evidence for this perturbation again shown here in this study [16]. In terms of physical performance, it would appear that the physiological response of the participants was driven by the unaccustomed hot and humid environment, more so than the time-of-day differences. In this study, athletes routinely trained throughout the solar day and appear to entrain systems to adapt to negate the circadian influence often seen in the morning. The questions to be posed therefore are the given differential diurnal response of the respective variables measured in this study: does this relate to a differential diurnal physiological/immune stress response? Importantly, does this result in a diurnal training effect phase response? Given the complexities of the systems involved in these responses, these will be difficult questions to answer but merit further research. A differential training response depending on the time of day, if elucidated, could be very useful in terms of training and training time efficiencies.

Hypothesis 1 was therefore rejected as there were no significant differences in the running performance of the athletes between the morning and evening trials; therefore, we believe to the habitual training patterns of the athletes and additionally to the masking effect of the unaccustomed environmental conditions of heat and humidity. We did, however, see some differential responses in the physiological and immunological variables depending on time of day, although not across the entire panel of measures, and hence, hypothesis 2 was therefore partially accepted.

### 4.1. Practical Application of Results

The current data suggest that exercise and/or time of day would interfere with clinical endocrine profiling of a population exercising in the evening. There is evidence that morning exercise exerts higher physical demands on the individual [35]. Hence, athletes and coaches should take into consideration these phenomena when planning intensity training or racing in the morning time. In addition, athletes may endeavour to adjust their internal clock to its peak performance time, regardless of what clock time the athletic event is occurring. Coaches are required to design training schedules to either enhance or avoid obvious issues when competing in uncustomised environmental conditions. Thus, from a clinical perspective, the morning HR may be a cause for concern for the risk of cardiovascular events, where it is reported to be more pronounced in the morning hours compared to the rest of the day, especially when one additionally considers the results found here, for nonacclimatised participants exercising in hot and humid conditions. Furthermore, athletes are advised to train at the same time as planned races to advance and shift their circadian rhythm and minimise physiological strain and immunological suppression.

The findings of this research not only apply to athletes engaging in strenuous endurance events but also may apply to the general population, where the stress of physical exertion is combined with environmental stress at different times of the day, or even in some occupations, for example, firefighters.

Despite our findings, we acknowledge a number of limitations. Firstly, within the laboratory setting of this study, participants were exposed to the hot and humid condition only during a limited time. Secondly, we are unable to determine the exact mechanism underlying our findings due to complex and interrelated interactions. Measuring other hormonal variables such as cortisol and melatonin would help bring clarity to these results, elucidating further the prevailing mechanisms causing the high morning physiological strain and evening immune suppression.

## Figures and Tables

**Table 1:** Stature and body mass in both trials.

	09:00 hs		18:00 hs	
	Pretrial	Posttrial	Pretrial	Posttrial
Body mass (kg)	71.80 ± 7.13	70.78 ± 7.00	71.55 ± 7.04	70.76 ± 6.91
Body water loss (kg)	1.02 ± 0.13		0.76 ± 0.13	
Stature (cm)	180.96 ± 5.38^∗^		179.50 ± 4.97^∗^	

∗ denotes a significant diurnal difference. Body water loss = pretrial body mass-posttrial body mass.

**Table 2:** The effect of the exercise trial in heat and circadian rhythmicity on HR, RPE, and speed at 09:00 hs and 18:00 hs.

	09:00 hs	18:00 hs	% difference
Mean speed (km·h^−1^)	14.75 ± 0.43	14.80 ± 0.45	0.3%
Resting HR (beats·min^−1^)	50 ± 7	53 ± 7	6%
Peak HR (beats·min^−1^)	182 ± 11	181 ± 13	0.5%

Values are mean ± SD.
